# Use of a matrix for apexification procedure with mineral trioxide aggregate

**DOI:** 10.4103/0972-0707.62629

**Published:** 2010

**Authors:** Roheet A Khatavkar, Vivek S Hegde

**Affiliations:** Department of Conservative Dentistry and Endodontics, MA Rangoonwala Dental College, Pune, India

**Keywords:** Apexification, artificial barrier, calcium sulfate, mineral trioxide aggregate

## Abstract

This articles describes a technique for placement of a matrix barrier prior to use of mineral trioxide aggregate (MTA) as an artificial root-end barrier. The technique also demonstrates the use of a delivery system utilizing large-bore needles for the predictable and precise placement of the barrier materials at the apex of the tooth.

## INTRODUCTION

Recently, mineral trioxide aggregate (MTA) has been popularized in endodontics due to a large amount of research indicating the beneficial properties of the material in terms of bio-compatibility, ease of manipulation and placement, and a wide array of applications.[1–5] It has been used for procedures ranging from direct pulp capping to perforation repair as well as for inducing an artificial barrier in open-apex cases (apexification).[6,7] A study comparing the effectiveness of calcium hydroxide versus MTA has shown that this material has been found to be as effective as calcium hydroxide (Ca(OH)_2_) for the treatment of cases with an open apex. MTA has shown to be effective in performing the same procedure in a considerably lesser period of time with predictable results. The authors also concluded that the chances for biological calcific bridge formation are favorable when the root canal apices are flush or underfilled with MTA.[8]

Lemon advocated the use of a matrix when the perforation diameter is larger than 1 mm to avoid extrusion of the sealing material.[9] The use of a matrix is advisable since its placement in the area of bone destruction provides a base on which the sealing material, especially MTA, can be placed and packed in the perforation.[9–13] Several materials have been recommended to create a matrix, in cases of perforations as well as teeth with incomplete formation of apex; including calcium hydroxide, hydroxyapatite, resorbable collagen and calcium sulfate.[11–16]

The use of calcium sulfate or calcium sulfate hemihydrate in combination with collagen has been recommended as a barrier in a number of studies. This article presents a detailed case report of the use of such a matrix prior to placement of MTA as an apical barrier.

## CASE REPORT

**Figure 1:**
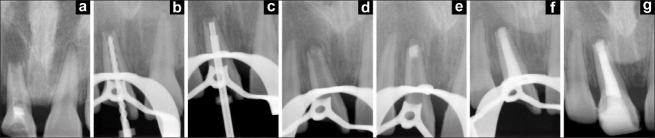
Series of radiographs showing placement of the barrier material and mineral trioxide aggregate. (a) Pre-operative radiograph showing remnants of calcium hydroxide paste; (b) 140 no. K-file placed to working length; (c) System of needles used as a carrier for placement of the barrier material and mineral trioxide aggregate; (d) Barrier material in place in area of the bony lesion; (e) Mineral trioxide aggregate compacted against the barrier material; (f) Backfill performed using thermoplasticized Gutta-percha in the next appointment; (g) Completed coronal restoration

An 18-year-old male patient reported to the Department of Conservative Dentistry and Endodontics, M.A. Rangoonwala Dental College, with a chief complaint of a discolored maxillary right central incisor. History revealed that the patient had suffered trauma at the age of 8 years and had received endodontic treatment from a private clinic. Clinical examination revealed discolored tooth no. 11, and access cavity preparation was performed in the tooth. Radiographic examination revealed Ca(OH)_2_ paste in the canal and an incomplete formation of the root apex [Figure 1]. After the placement of rubber dam, the pulp chamber was cleaned and irrigated with 2.5% sodium hypochlorite. The width of the canal was gauged, and it was found to be equal to an ISO 140 no. K-file (Beutelrock, München, Germany). The walls of the canal were cleaned using a circumferential filing motion followed by an intracanal dressing with Ca(OH)_2_ paste and sealing of the access cavity with Cavit-G (ESPE, Seefeld, Germany).

**Figure 2:**
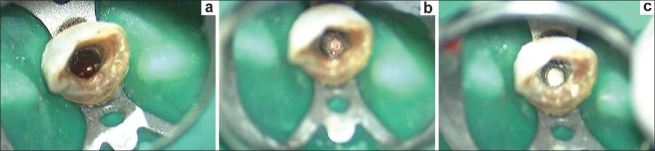
Snapshots taken under the dental operating microscope at ×8 magnification; (a) Bleeding present at the apical exit of the foramen; (b) Calcium sulfate apical barrier in place; (c) Mineral trioxide aggregate compacted against the apical barrier

**Figure 3:**
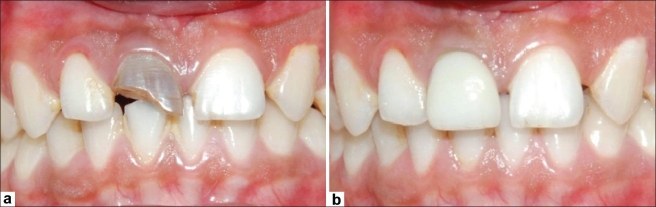
(a) Pre-operative photograph showing discolored maxillary right central incisor; (b) Post-operative photograph showing tooth restored with an all-ceramic crown

One week later, the tooth was again isolated under rubber dam and the canals thoroughly irrigated with saline to wash out any remnants of the Ca(OH)_2_ dressing and 17% liquid EDTA Smear Clear (SybronEndo, CA, USA) for removal of the smear layer [Figure 2]. A combination of calcium sulfate hemihydrate and de-mineralised bone particles (Type-I collagen) in powdered form, Osseomold (Advanced Biotech Products, India) was used in the formation of the artificial barrier. The powder was mixed with saline placed and packed using a delivery system comprised of 2 large bore needles. The material was packed against the bone and was allowed to be pushed beyond the apex into the bony space formed due to the periapical lesion in order to achieve a matrix for the placement of MTA. Any excess material left in the canal was removed using the ISO 140 no. K-File. A radiograph was taken to confirm the placement of the barrier followed by mixing the White MTA-Angelus (Angelus, Londrina, PR, Brazil) and using the same system of needles for delivery of the material. Following the placement of MTA over the barrier, butt-end of a paper point was used to compact the material and clear out any excess from the walls. Moistened gauze was placed in the remainder of the canal and the access cavity sealed using glass ionomer cement (Fuji, *GC* Corporation, Tokyo, Japan). Since the MTA takes around 6-8 hours for complete setting, the patient was called on the next day and the moist gauze was removed and a plugger was used to check the consistency of the MTA and to examine if the material was thoroughly set. Subsequently, backfill was performed using Obtura (Obtura/Spartan, Fenton, MO, USA), and the access cavity was sealed using composite resin. A radiograph confirmed the completion of the endodontic therapy. Two weeks recall radiograph revealed the complete resorption of the calcium sulfate barrier. The patient was advised a full coverage restoration. Since the patient demanded better esthetics for the anterior tooth, we opted for a metal-free crown. Accordingly, an *IPS* E.Max^®^ (Ivoclar Vivadent, AG, Germany) all ceramic crown was fabricated and cemented using RelyX-100 cement (3M ESPE, Germany). A 3-month follow-up revealed complete healing and bone formation [Figure 3].

## DISCUSSION

A number of methods have been recommended for artificial apical closure. For a long time, calcium hydroxide has been used as an apexification material. The newly developed MTA has also been shown to be effective in artificial root-end closure. Both materials were found to stimulate the formation of mineralized tissue, thereby providing an adequate seal in the apical region.[17] MTA, a bio-compatible material, can be used to create a physical barrier that also helps in formation of bone and periodontium around its interface.[1–5] Although earlier studies recommended the use of a Ca(OH)_2_ prior to MTA placement, recent studies report that the initial use of Ca(OH)_2_ paste was not necessary for apexification to occur, and it has shown to be strongly related to the extrusion of MTA and formation of barriers beyond the limits of the root canal walls.[3] The major problem in cases of a wide open apex is the need to limit the material to the perforation, thus avoiding the extrusion of a large amount of material into the periodontal tissue.[18,19]

A large volume of the extruded material may set before it disintegrates and get resorbed. This might result in the persistence of the inflammatory process, which may complicate or even prevent repair of the tissue.[18,19] Using a matrix avoids the extrusion of the material into the periodontal tissues, reduces leakage in the sealing material and allows favorable response of the periodontal tissues. As mentioned before, a number of materials are available for the formation of the apical matrix.

Some studies mention the use of small pieces of collagen membranes that are packed within the bone space to create a matrix against which MTA can be packed. This method seems to be technique-sensitive requiring a high level of accuracy in positioning and placement of these membranes.[12,13]

**Figure 4:**
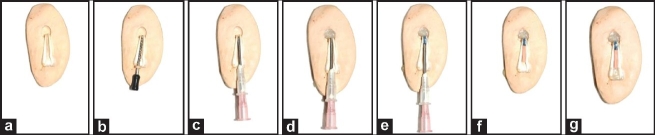
A vertically sectioned model demonstrating the technique for the placement of the apical barrier and MTA. (a) Pre-operative condition of a tooth with a wide open apex; (b) K-file placed to gauge the diameter of the apical exit of the canal; (c) A system of two large-bore needles in place for placing the material directly in the apical region; (d) Barrier material (white colored) placed and pushed in the bony lesion; (e) Mineral trioxide aggregate (blue colored) placed against the barrier material at a thickness of 4-5 mm; (f) Thermoplasticized Gutta-percha compacted to the orifice level; (g) Composite resin used to seal the coronal access preparation

The technique utilizing calcium sulfate or a combination of calcium sulfate and collagen in a powdered form is relatively simple as the placement is similar to that done for MTA through the use of a carrier device. Further, any excess material is easily removed by instrumenting the apical region.[13–15] The sequence of steps followed in this technique have also been demonstrated using an artificial model [Figure 4].

Calcium sulfate was found to induce tissue repair when it was used for filling large surgical cavities because invagination of the epithelium, which prevents bone formation, is avoided. Calcium sulfate is resorbed after about 4 weeks, thereby assisting in the formation of new bone tissue and more favorable repair.[20–22]

Calcium sulfate may also be introduced using specialized devices such as the Messing Gun or Dovgan Carriers. Considerable care is taken to ensure that the calcium sulfate does not contaminate the walls of the canal as it can interfere with the close adaptation of MTA. Ideally, the tip of the delivery syringe should reach beyond the apical aperture. Calcium sulfate is placed in small increments, and the placement is confirmed radiographically. It has a radio-opacity that is similar to dentin, and it sets within 1-2 min. The speed of setting of the calcium sulfate mandates that the tip of the carrier be cleaned as soon as possible to avoid the tube being blocked by the set material.

The combination of calcium sulfate as a matrix and MTA has been demonstrated to be a good option for creating artificial root-end barriers. The placement of MTA is predictable and easily achieved, and the outcomes are very encouraging.
